# Fully automated quantification of cardiac chamber and function assessment in 2-D echocardiography: clinical feasibility of deep learning-based algorithms

**DOI:** 10.1007/s10554-021-02482-y

**Published:** 2022-02-13

**Authors:** Sekeun Kim, Hyung-Bok Park, Jaeik Jeon, Reza Arsanjani, Ran Heo, Sang-Eun Lee, Inki Moon, Sun Kook Yoo, Hyuk-Jae Chang

**Affiliations:** 1 CONNECT-AI Research Center, Yonsei University College of Medicine, Seoul, South Korea; 2 Graduate Program of Biomedical Engineering, Yonsei University College of Medicine, Seoul, South Korea; 3 Department of Cardiology, Catholic Kwandong University International St. Mary’s Hospital, Incheon, South Korea; 4 Department of Cardiovascular Diseases, Mayo Clinic Arizona, Phoenix, AZ USA; 5 Department of Cardiology, Hanyang University Seoul Hospital, Hanyang University College of Medicine, Seoul, South Korea; 6 Department of Cardiology, Ewha Womans University Seoul Hospital, Seoul, South Korea; 7 Division of Cardiology, Department of Internal Medicine, Soonchunghyang University Bucheon Hospital, Bucheon, South Korea; 8 Department of Medical Engineering, Yonsei University College of Medicine, 50-1 Yonsei-ro, Seodaemun-gu, Seoul, 03722 South Korea; 9 Division of Cardiology, Department of Cardiology, Severance Cardiovascular Hospital, Yonsei University College of Medicine, Yonsei University Health System, 50-1 Yonsei-ro, Seodaemun-gu, Seoul, 03722 South Korea; 10 Ontact Health Co., Ltd., Seoul, South Korea

**Keywords:** Echocardiography, Deep learning, Fully automated

## Abstract

We aimed to compare the segmentation performance of the current prominent deep learning (DL) algorithms with ground-truth segmentations and to validate the reproducibility of the manually created 2D echocardiographic four cardiac chamber ground-truth annotation. Recently emerged DL based fully-automated chamber segmentation and function assessment methods have shown great potential for future application in aiding image acquisition, quantification, and suggestion for diagnosis. However, the performance of current DL algorithms have not previously been compared with each other. In addition, the reproducibility of ground-truth annotations which are the basis of these algorithms have not yet been fully validated. We retrospectively enrolled 500 consecutive patients who underwent transthoracic echocardiogram (TTE) from December 2019 to December 2020. Simple U-net, Res-U-net, and Dense-U-net algorithms were compared for the segmentation performances and clinical indices such as left atrial volume (LAV), left ventricular end diastolic volume (LVEDV), left ventricular end systolic volume (LVESV), LV mass, and ejection fraction (EF) were evaluated. The inter- and intra-observer variability analysis was performed by two expert sonographers for a randomly selected echocardiographic view in 100 patients (apical 2-chamber, apical 4-chamber, and parasternal short axis views). The overall performance of all DL methods was excellent [average dice similarity coefficient (DSC) 0.91 to 0.95 and average Intersection over union (IOU) 0.83 to 0.90], with the exception of LV wall area on PSAX view (average DSC of 0.83, IOU 0.72). In addition, there were no significant difference in clinical indices between ground truth and automated DL measurements. For inter- and intra-observer variability analysis, the overall intra observer reproducibility was excellent: LAV (ICC = 0.995), LVEDV (ICC = 0.996), LVESV (ICC = 0.997), LV mass (ICC = 0.991) and EF (ICC = 0.984). The inter-observer reproducibility was slightly lower as compared to intraobserver agreement: LAV (ICC = 0.976), LVEDV (ICC = 0.982), LVESV (ICC = 0.970), LV mass (ICC = 0.971), and EF (ICC = 0.899). The three current prominent DL-based fully automated methods are able to reliably perform four-chamber segmentation and quantification of clinical indices. Furthermore, we were able to validate the four cardiac chamber ground-truth annotation and demonstrate an overall excellent reproducibility, but still with some degree of inter-observer variability.

## Introduction

Echocardiography is a primary cardiac imaging tool which provides noninvasive real-time imaging for identifying cardiac structure along with function in clinical practice. However, unlike other imaging modalities such as computed tomography (CT) or magnetic resonance imaging (MRI), echocardiography is highly operator dependent with various types of artifacts which can occur during image acquisition as well as post-processing [1]. Unavoidably, these limitations can cause large inter and intra observer variability and poor reproducibility, resulting in clinical shortcomings, in particular, when it comes to monitoring longitudinal quantitative measurements [2, 3]. Even though, semi-automated delineation and quantification of cardiac structures have demonstrated their utility in the quantitative echocardiographic assessment, they still need significant amount of manual modification which is time-consuming and may introduce the risk of the inter-/intra-observer variability [4].

The adoption of artificial intelligence (AI) technology in echocardiographic imaging has recently emerged as a novel alternative solution for these challenges [5, 6]. With the advance of deep neural networks, there have been attempts to develop fully-automated algorithms for mainly focusing on the left ventricle (LV), where there is a clinical unmet need for the accuracy and reproducibility of quantitative assessments [7]. Importantly, in order to develop an accurate chamber segmentation deep learning algorithm, which is the basis of the functional assessment, is dependent on the ground-truth image data, being so called ‘training data set’, being essentially accurate and reproducible [8, 9].

The poor repeatability is primarily due to the low signal-to-noise ratio, edge dropout, and presence of shadow, there is a high variability in quantitative assessment of echocardiography [2, 3]. However, to date, there has been no investigation of the inter-/intra-observer variability of ground-truth of four chamber data itself which is crucial for the accurate and advanced deep-learning algorithms. In addition, there have been no comparisons among currently available deep learning algorithms to suggest the most potent approaches for fully automated quantitative analysis nor have there been clinical performance comparisons made between the algorithms and conventional techniques.

Therefore, firstly we sought to evaluate whether currently prominent deep learning methods could accurately estimate clinical indices including LV mass, LV/LA volume, and EF when compared mutually as well as with conventional methods. Secondly, we validated the reproducibility of our manually created ground-truth segmentations in two-dimensional echocardiography which are imperative for the development of highly advanced deep-learning AI based algorithms.

## Methods

### Study population

In this study, we retrospectively enrolled consecutive patients who had visited our cardiology outpatient clinic and underwent transthoracic echocardiogram (TTE) due to various symptoms from December 2019 to December 2020. The Institutional Review Board at Yonsei University Severance Cardiovascular Hospital approved this study protocol. Inclusion criteria consisted of having had all echocardiography visually correspond to standard views and we excluded patients with diagnosis of heart failure, coronary artery disease, valvular heart disease, known pregnant state, uninterpretable quality of echocardiographic images or images that were acquired by non-standardized scan angles, and those had abnormal echocardiograms (Table 1). All patients were scanned in the left lateral position using grayscale second-harmonic 2D imaging techniques, with the adjustment of image contrast, frequency, depth, and sector size for adequate frame rate and optimal LV border visualization. All patients received a complete quantification report, which was validated by cardiologists. Image quality was defined based on percent endocardial border visualization as good image quality: 67%–100%, fair image quality: 34%–66%, and poor image quality: 0%–33% [10]. Echocardiographic images were acquired by standard ultrasound equipment including Vivid 9 (GE Healthcare, Horton, Norway; n = 259), EPIQ 7C (Phillips Healthcare, Andover, MA, USA; n = 170), Acuson SC2000 (Siemens, Mountain View, CA, USA; n = 42), and Artida (Canon Medical Systems, Tokyo, Japan; n = 29).

**Table 1:** Baseline clinical characteristics (n = 500)

	n
Age (years)	36.2 ± 12.6
Male, n(%)	251 (50.2)
Height (cm)	167.1 ± 8.6
Weight (kg)	62.2 ± 11.3
Body mass index (kg/m^2^)	22.2 ± 2.9
Body surface area	1.7 ± 0.19
Systolic BP (mmHg)	119.4 ± 15.3
Diastolic BP (mmHg)	74.2 ± 11.4
Hypertension, n(%)	215 (43)
Diabetes, n(%)	110 (22)
Dyslipidemia, n(%)	235 (47)
Vendors, n(%)	
GE Healthcare	259 (51.8)
Philips Healthcare	170 (34.0)
Siemens	42 (8.4)
Canon Medical Systems	29 (5.8)
LVEF	67.0 ± 4.9
LV mass	132.8 ± 32.7
LA volume	43.3 ± 10.7
LA volume index	25.4 ± 5.4

### Ground-truth generation

**Fig. 1:**
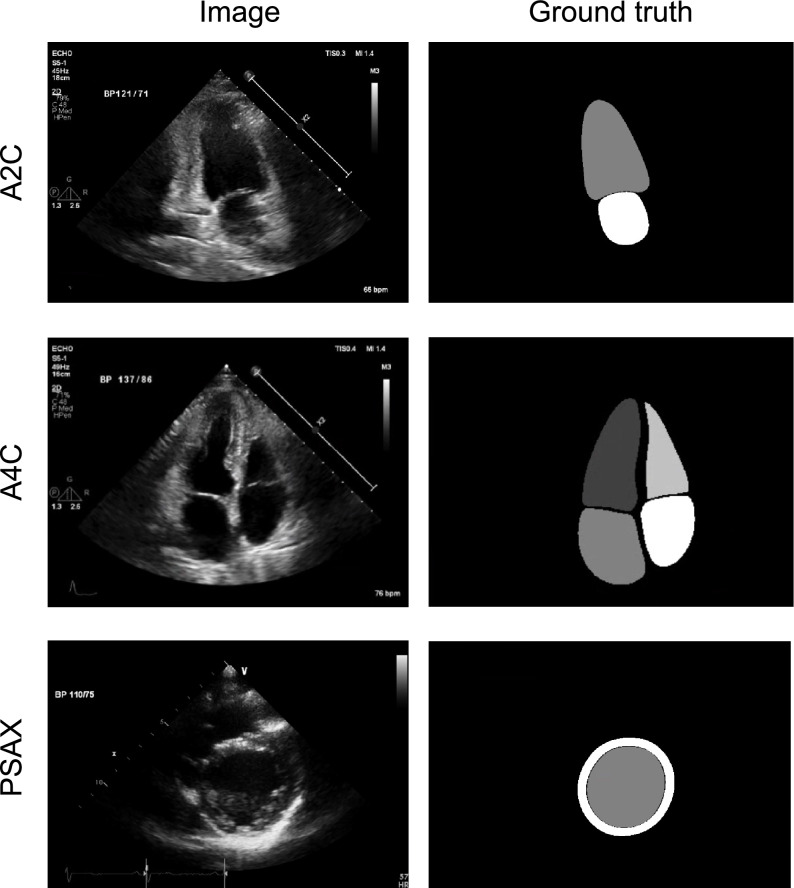
Representative echocardiography images and segmented cardiac chambers by experts

Anonymized echocardiographic digital images were analyzed and annotated in a core laboratory, where experienced sonographers (five expert sonographers who all had more than 5 years of echocardiographic experience) manually contoured cardiac structures according to the recommendations of the American Society of Echocardiography [11]. For each patient, we selected a set of B-mode images including apical two chamber (A2C) view, apical four chamber (A4C) view and parasternal short axis (PSAX) view at papillary muscle (PM) level which included at least one cardiac cycle. In a series of multi-video frames, manual annotation at end-diastole (ED) and end-systole (ES) for four chambers were performed using a commercial annotation tool (OsiriX, Pixmeo, Switzerland). Two chambers including LV and left atrium (LA) were delineated when available in A2C, A4C, and PSAX views, respectively. In addition, we further delineated four chamber structures including right ventricle (RV) and right atrium (RA) in A4C view (Fig. 1). During LV endocardial wall segmentation, trabeculations and papillary muscles were excluded in PSAX view at PM level.

### Inter- and intra-observer reproducibility analysis

To validate our manually created ground truth annotation, we performed both inter- and intra- observer variability analysis. Two sonographers, who both had more than 5 years of echocardiography experience with more than 5000 echocardiographic examinations, were chosen and the variability of clinical indices were assessed on a randomly selected echocardiographic images of A2C, A4C, and PSAX views in 100 patients.

One sonographer manually annotated LV, LA, RV, and RA contours on the three views at ED and ES with an interval of one month with a randomly shuffled analysis preventing the observer from being influenced by previous measurements (intra-observer variability). The other sonographer annotated the same groups without being influenced by the other (inter-observer variability).

### Dataset splitting

A total of 500 echocardiograms were allocated for developing automated deep learning methods. Each of the echocardiograms consisted of consecutive frames with dozens of still images. All information was removed from echocardiograms that could identify individual patients. Echocardiographic images were extracted from anonymized DICOM files, and unorganized videos with different views were grouped according to their views. The entire dataset containing 500 patients was divided into training (80%, n = 400), validation (10%, n = 50), and test set (10%, n = 50) for deep learning methods. The fivefold cross validation was employed to analyze the generalization performance of ML methods. The entire dataset containing 500 patients was divided into five groups. In training stages, four subsets were used for training and validation of the network. In test stages, the remainder was employed to evaluate the ML model.

### Deep learning-based algorithms

**Fig. 2:**
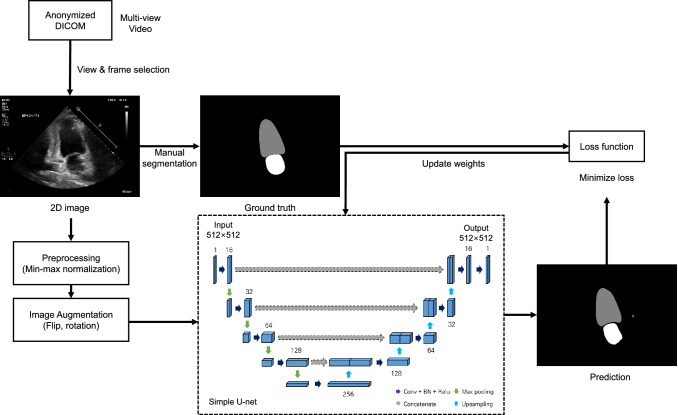
Schematic workflow of the convolutional neural network architecture

To automatically segment cardiac structures in echocardiography, we employed three deep learning models based on U-net which were used for biomedical image segmentation and have demonstrated high performance on segmentation of organs [12]. The U-net consists of a fully convolutional encoder path, called backbone with a symmetric expanding decoder path for segmentation. We constructed three deep learning models on the basis of encoder-decoder architecture of U-net. In our experiments, we deployed the backbone of U-net architecture with residual and dense blocks, which shows robust performance in the image classification network with a U-net decoder path [13, 14]. The flow chart of deep learning methods is shown in Fig. 2.

### Training strategy

Given input images and corresponding annotated masks containing different categories with their views, data augmentation was performed with up-down, left–right flips, and rotation. For training deep learning models, a pixel-wise cross-entropy loss function was employed to minimize inaccuracy of prediction. An Adam optimizer with learning rate of 0.0001 was adopted to optimize network parameter. We trained our models from scratch without using any pretrained weight for initialization. We randomly shuffled training dataset and trained deep network for 200 epochs with a mini-batch size of 5. All input cases were resized to 512 × 512 due to GPU memory limitation and then the image intensity values were normalized into the range of [0,1].

### Performance analysis of deep-learning models

Dice similarity coefficient (DSC) and IOU, and clinical indices such as volume, mass, and EF were included to compare the performance of deep learning methods. The DSC and IOU are both quantifies the pixel-wise degree of similarity between the model-predicted segmentation mask and the ground truth, and ranges from 0 (no similarity) to 1 (identical). We employed segmentation results of the deep learning model to calculate clinical index to compare chamber structure quantification and EF based on standard guidelines. Left and right volume were calculated by the area-length method derived by the long axis and area of A2C and A4C [11]. LVM was calculated as the left ventricular myocardial volume derived by the delineation of its endocardial and epicardial borders and multiplied with the specific gravity of myocardial tissue (assuming a tissue density of 1.05 g/ml). These annotations, which were established and verified by board-certified cardiologists, were used as the ‘ground truth’ for the deep-learning model.

### Statistical analysis

Continuous and normally distributed variables were represented as mean ± SD and median with interquartile range (IQR) for non-normally distributed variables. Comparison between ground truth and prediction results were assessed by the paired t test and Pearson correlation coefficient using two-sided p values. A p value of < 0.05 was considered significant. Bland–Altman plots with 95% confidence intervals for correlation were calculated. Inter-observer and intra-observer reproducibility were assessed by the intraclass correlation coefficients (ICC) for absolute agreement of single measures between two observers. All statistical analyses were performed using commercially available statistics software (MedCalc, version 18.9, MedCalc software Inc., Mariakerke, Belgium).

## Results

### Baseline patient characteristics

Baseline characteristics of subjects are described in Table 1. The mean age was 36.2 ± 12.6 years, and 50.2% of the population were male. The mean Body mass index was 22.2 ± 2.9, and Body surface area was 1.7 ± 0.19. The average of LVEF was 67.0 ± 4.9 and LV mass was 132.8 ± 32.7, and LA volume was 43.3 ± 10.7, respectively. Among patients analyzed, 215 (43%) had hypertension, 110 (22%) had diabetes, and 235 (47%) had dyslipidemia. The patients’ echocardiographic were obtained using various vendors including GE Healthcare (51.8%), Philips Healthcare (34.0%), Siemens (8.4%), and Canon Medical Systems (5.8%).

### Performance comparison between deep-learning models

**Fig. 3:**
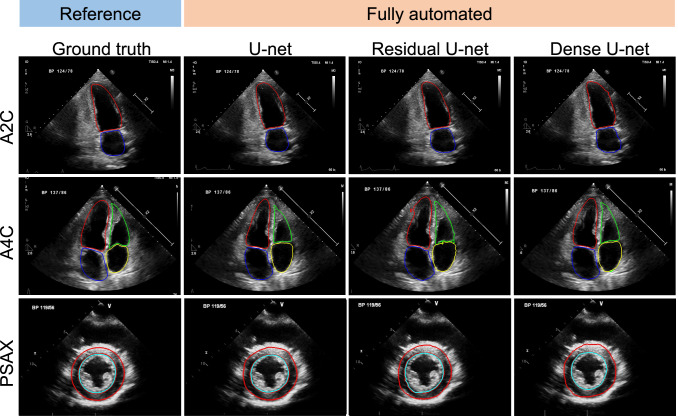
Representative segmentation results of fully automated methods

In Table 2, automated segmentation performances between deep neural networks (U-net vs. Res-U-net vs. Dense-U-net) were compared by using DSC and IOU metrics. Except LV wall area on PSAX PM level view (average DSC of 0.83, IOU 0.72), all deep learning methods showed an overall excellent performance (average DSC 0.91 to 0.95 and average IOU 0.83 to 0.90) (Table 2 and Fig. 3). There were no significant differences observed between the three deep learning methods for all the parameters analyzed at each echocardiographic view (Table 2).

**Table 2:** Performance of each deep neural network (rows) in A4C and PSAX

View	Segmented area	Metric	U-net	Res-U-net	Dense-U-net	Avg.
A4C	LV cavity	DSC	0.96	0.95	0.92	0.94
		IOU	0.92	0.91	0.86	0.90
	LA cavity	DSC	0.94	0.92	0.95	0.94
		IOU	0.88	0.86	0.91	0.88
	RV cavity	DSC	0.91	0.91	0.90	0.91
		IOU	0.87	0.86	0.83	0.85
	RA cavity	DSC	0.93	0.92	0.92	0.92
		IOU	0.84	0.83	0.83	0.83
A2C	LV cavity	DSC	0.95	0.95	0.95	0.95
		IOU	0.90	0.90	0.90	0.90
	LA cavity	DSC	0.93	0.92	0.92	0.92
		IOU	0.87	0.85	0.85	0.86
PSAX	LV cavity	DSC	0.95	0.95	0.95	0.95
		IOU	0.90	0.91	0.91	0.90
	LV myocardium	DSC	0.83	0.82	0.84	0.83
		IOU	0.72	0.71	0.73	0.72

Error measures described as mean and std computed between ground truth and results

**Fig. 4:**
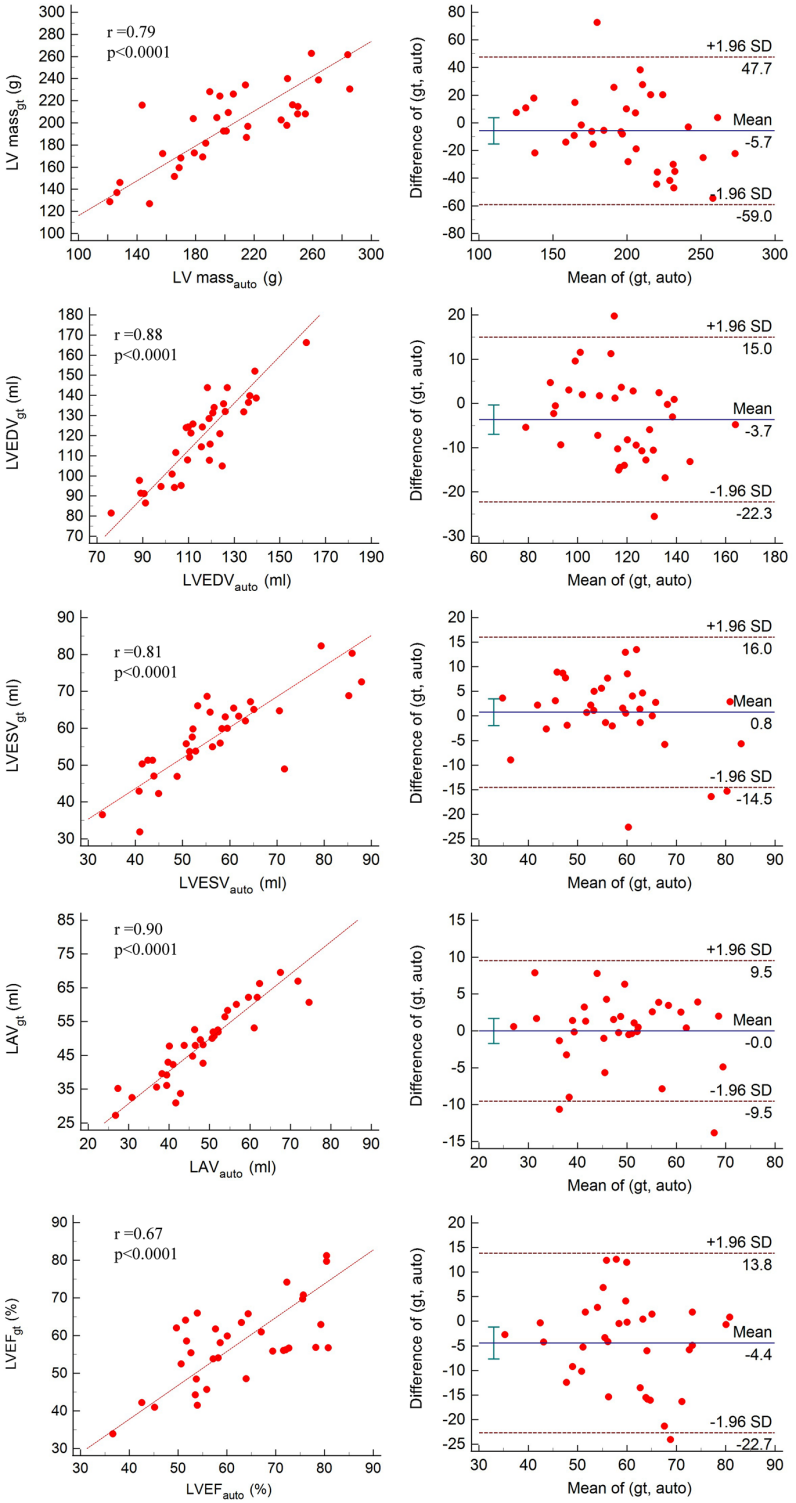
Comparison between automated method and manual measurement of LV volumes, mass, LA volume, and EF: linear regression (left) and Bland–Altman analysis (right) for LV mass, LVEDV, LVESV, LAV, and LVEF from top to bottom

We also compared commonly used clinical indices such as LAV, LVEDV, LVESV, LV mass, and EF. There were no significant differences found between ground truth and automated ML measurements (Table 3). The correlation and Bland–Altman plots for these indices are presented in Fig. 4. Overall, the model prediction for all structures correlated well with no significant differences as compared with manual ground truth annotation: LAV (r = 0.90; p < 0.001), LVEDV (r = 0.88; p < 0.001), LVESV (r = 0.81; p < 0.001), LV mass (r = 0.79; p < 0.001), and EF (r = 0.67; p < 0.001). Bland–Altman analysis demonstrated a mean difference, especially in EF, which is the ratio of LVEDV and LVESV, showing that accumulation of variability resulted in high variability. However, the difference between manual and automated analysis were not statistically significant.

**Table 3:** Comparison of clinical indices between automated and ground truth from two-dimensional echocardiography

	Median value (IQR)		*p*
	Ground truth	Automated ML (simple U-Net)	
Left atrium volume, ml	48.01 (40.02–54.48)	48.90 (42.91–52.55)	0.3172
Left ventricle diastolic volume, ml	121.11 (105.22–128.42)	117.21 (104.45–125.41)	0.2628
Left ventricle systolic volume, ml	55.48 (48.86–63.31)	58.75 (51.33–65.12)	0.1584
Left ventricle mass, g	199.98 (169.84–242.86)	203.43 (172.24–224.34)	0.1741
Ejection fraction	61.60 (51.50–71.56)	56.82 (52.46–63.43)	0.0797

**Fig. 5:**
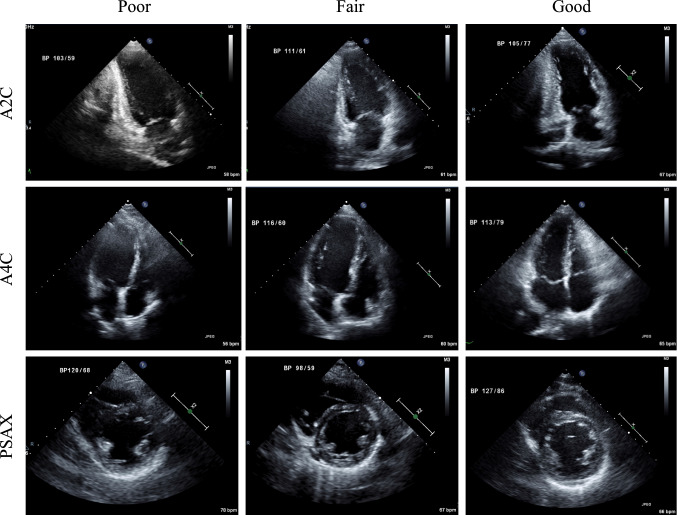
Representative echocardiography image from good to bad image quality

On the other hand, automated deep learning algorithm showed lower performance generally in those with ‘poor’ image quality as compared to ‘good’ or ‘fair’, especially in LV myocardium (poor 0.70 vs. fair 0.83 vs. good 0.84) and LV cavity (poor 0.87 vs. fair 0.94 vs. good 0.94) (Table 4 and Fig. 5).

**Table 4:** Comparison of performance with image quality in DSC

View	Segmented area	Poor	Fair	Good	Avg.
A2C	LV cavity	0.87	0.94	0.94	0.94
	LA cavity	0.82	0.92	0.89	0.95
A4C	LV cavity	0.95	0.93	0.96	0.97
	LA cavity	0.93	0.90	0.92	0.91
	RV cavity	0.85	0.87	0.90	0.92
	RA cavity	0.91	0.89	0.91	0.92
PSAX	LV cavity	0.91	0.95	0.95	0.93
	LV myocardium	0.70	0.83	0.84	0.83

**Fig. 6:**
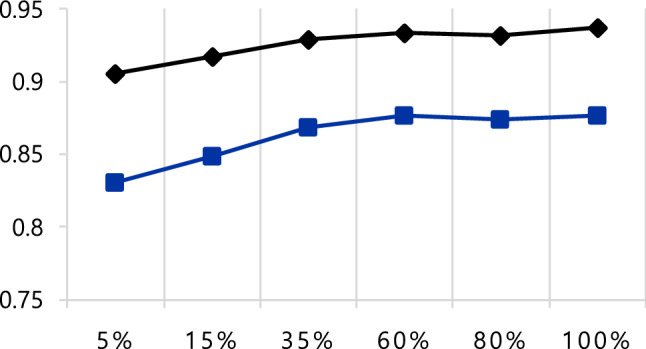
Effect of the number of training dataset for segmentation of echocardiography [black denotes dice similarity coefficient (DSC), Blue indicates intersection over union (IOU)]

Additionally, we tested the impact of the number of training dataset for robustness of ML algorithm. There were linear association between the performance of automated ML analysis and the increment of dataset until 60% of the training dataset (n = 300; 1,271 frames) achieving already the maximal DSC and IOU of 0.93 and 0.87. After this point, the accuracy was sustained regardless of the number of training dataset (Fig. 6).

### Inter- and intra-observer variability of ground truth annotation

Intra observer variability highly correlated for all of the measurements as follows: left atrium volume: ICC = 0.995, p < 0.001; LV diastolic volume; ICC = 0.996, p < 0.001, LV systolic volume; ICC = 0.997, p < 0.001, left ventricular mass; ICC = 0.991, p < 0.001; and EF; ICC = 0.984, p < 0.001 (Table 5). Inter observer variability showed slightly lower correlation compared to intra observer variability, as follows: left atrium volume: ICC = 0.976, p < 0.001; LV diastolic volume; ICC = 0.982, p < 0.001, LV systolic volume; ICC = 0.970, p < 0.001, left ventricular mass; ICC = 0.971, p < 0.001; and EF; ICC = 0.889, p < 0.001). The variability of EF, which is the difference ratio between LVEDV and LVESV, showed the lowest correlation among clinical metrics (Table 5).

**Table 5:** Reproducibility of the analysis of each structure

Clinical index	Intra observer variability (ICC) (95% CI)	*p*	Inter observer variability ICC (95%CI)	*p*
Left atrial volume	0.995 (0.993–0.997)	< 0.001	0.976 (0.964–0.984)	< 0.001
Left ventricle end diastolic volume	0.996 (0.993–0.997)	< 0.001	0.982 (0.973–0.988)	< 0.001
Left ventricle end systolic volume	0.997 (0.995–0.998)	< 0.001	0.970 (0.955–0.980)	< 0.001
Left ventricular mass	0.991 (0.986–0.994)	< 0.001	0.971 (0.956–0.980)	< 0.001
Left ventricular ejection faction	0.984 (0.976–0.989)	< 0.001	0.899 (0.847–0.933)	< 0.001

*ICC* intraclass correlation, *CI* confidence interval

## Discussion

In this study, we were able to show that the current prominent deep learning-based methods can be reliably utilized for automated quantification of cardiac chambers in 2-D echocardiography with no significant segmentation performance variabilities between these algorithms. Simple U-net, Res-U-net, and Dense-U-net are currently the most prominent deep learning algorithms for image segmentation and have demonstrated their robust performance on organ segmentation in biomedical imaging [12–14]. However, we observed that the segmentation performance was substantially influenced by image quality especially involving certain parameters. In addition, we also validated the reproducibility of our manually created four chamber ground-truth annotations which are the fundamental basis of advanced deep learning algorithms, which had not been specifically investigated in prior studies. To our knowledge, this is the first study comparing current prominent deep learning algorithms and validating reproducibility of the four-chamber ground-truth. Furthermore, this is the largest manually annotated datasets acquired in different vendors which was then utilized for the evaluation of automated analysis of echocardiography.

A major well known limitation of quantitative analysis in clinical practice is the significant inter- and intra-observer variability involving tracing of endocardial borders [2, 3]. Even though semiautomated software was used, inherent low reproducibility impedes the reliable longitudinal quantitative assessment, particularly in the following situations: poor image quality scans, patients who have arrhythmias, complex abnormality in cardiac chambers such as congenital anomaly, multivalvular heart disease, presence of regional wall motion abnormalities in the LV due to myocardial infarction, and infiltrative myocardial diseases [15, 16]. In addition, the relatively time-consuming quantification process prohibits quantification of multiple frames or dynamic frame-by-frame analysis in order to average clinical measurements especially for patients with irregular heartbeats [17].

However, recently introduced fully automated deep learning-based cardiac chamber quantification techniques have no operator variability, providing almost real-time quantitative processing (0.12 s per frame with CPU), as well as enabling dynamic cardiac chamber quantification [15, 17]. Zhang et al. nicely demonstrated the feasibility and high diagnostic accuracy of U-Net deep learning-based segmentation algorithms and represented automated view classification with disease detection models, which could be a profound pipeline work for the realization of AI-based one button whole echocardiography quantitative analysis in the future [15]. However, in their study, segmentation and quantitative analysis performances were not tested in poor or modest image quality in various disease models which are frequently encountered in clinical practice. In addition, a relatively small number of ground-truth annotations were used at each view for training their deep learning algorithm from 124 to 214, which might not be sufficient for the development of robust segmentation algorithm. In addition, no validation of reproducibility for ground-truth was presented [15]. In our study, on the other hand, 500 ground-truth annotations were used in deep learning training and analyzed algorithm performances according to image quality. We found that in those with image quality, relatively low segmentation performance were observed for LV cavity, LA cavity, LV myocardium area, which is consistent with previously reported studies [15, 18, 19].

For the reliability of training data sets, we reported the interobserver and intra observer variability analysis from 100 patients (20% of total population), demonstrating that inter-observer variability was higher than intra-observer variability showing still some degree of variability exists between experienced experts. Another interesting finding was that high variance was shown in EF due to the accumulated error from EDLV and ESLV which has constantly shown in prior studies [15, 19]. Narang et al. introduced a ML based quantification of 3D echocardiography and compared it to cardiac MRI [17]. Although small number of patients (n = 20) were studied, their accurate and instant frame-by-frame LV and LA volume-time curve generation based on 3D segmentation has potential application for searching novel dynamic parameters predicting future outcome [17].

**Fig. 7:**
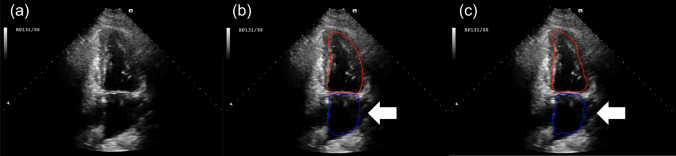
An example of worst prediction result. Left atrium with incomplete boundary interferes segmentation accuracy. The white arrow indicates incomplete left atrium boundary. **a** The original image, **b** ground truth overlaid on image, **c** prediction result overlaid on image (red: left ventricle, blue: left atrium)

The most significant barrier of deep learning method for echocardiography is the lack of large datasets. Recently, Arafati et al. [18] suggested additional adversarial training to fully convolutional networks to overcome data dependency of network and combined post processing procedure to improve the segmentation performance, but they also reported the variance of segmentation performance depending on age and gender in LV and LA segmentation, respectively. Thus, there are still several unsolved issues that need to be investigated for the full implementation of deep learning-based fully automated quantification into clinical practice. First, further advancement of deep learning based-segmentation algorithms should be made not only for relatively normal heart model but also for various disease models. These algorithms will also need to prove their robustness in studies with modest to poor image qualities (Fig. 7). Furthermore, similar to other imaging modalities, inherent operator variation of ground-truth itself is inevitable and the variability will naturally increase depending on image quality and the complexity of heart disease. Therefore, it is essential to diminish this variability and enhance the accuracy of segmentation algorithm through building up large number of accurately annotated ground-truth by experienced echocardiographic experts, despite is being time-consuming and labor-intensive.

In our study, we represented the relationship between the number of training datasets and segmentation performance showing that maximal performance level was already reached when 60% of the dataset was being utilized. Still, in order to establish that amount of datasets, it took 5 experienced experts working full time for three months. Moreover, to our knowledge, there has been no study up to now that has validated deep learning methods on the patients with different types of disease. Deep learning models can be improved by diverse sampled data without the class imbalance of disease types, which requires large databases to improve their robust accuracy [18, 20].

The recently emerged deep active learning (AL) method could be the solution for this unmet need for the clinical annotation process [21–23]. AL is a powerful method that enables data-efficient model. It reduces the laborious and expensive annotation tasks by carefully choosing data points for which ground-truth need to be labeled [24]. Specifically, AL selects the set of the most informative data points from a pool of unlabeled dataset, so it enables us to minimize the amount of data points that should be labeled and achieve comparable performance to a model trained with fully annotated dataset [25, 26].

Echocardiography has been established as an important primary screening test since the incidence of cardiovascular disease has been on the rise [27]. The non-invasive bedside approach enables clinicians to easily monitor the disease progression as well as the response to treatment [27, 28]. Furthermore, by using the serial assessment of quantitative parameters including global or longitudinal LV strain, it is possible to diagnose the disease at its preclinical stage and inhibit its progression [29–31]. The development of technology has allowed for the minimalization of echocardiographic systems such as portable handheld transthoracic echocardiography which can further broaden the utility of echocardiography in emergency settings by novice or non-expert paramedics [32–34]. However, the operator dependency of echocardiography particularly in achieving quantitative parameters serves as a significant limitation. Recently emerged deep learning-based AI technologies may allow us to level the playing field between rural and urban areas through AI aid or guided fully automated image acquisition and quantification. These novel methods can also be widely adopted in busy echocardiographic laboratories as well as be deployed in emergency departments for more efficient ways to triage patients.

### Study limitations

Our study has several limitations. These data should be interpreted cautiously because of its retrospective single-center study design; hence, further prospective multi-center studies are warranted. In addition, various types of disease models were not included in our analysis. However, for the first time, we evaluated the performance of fully automated deep learning-based methods according to image quality as well as the required number of training datasets to achieve the maximal performance. Therefore, our study results provided important considerations for future studies for developing specific disease model algorithms such as hypertrophic cardiomyopathy, and heart failure with reduced EF or having regional wall motion abnormality. Finally, we only included four chambers on limited echocardiographic views in our analysis. However, to establish a fully automated quantification, beyond the four cardiac chambers, aorta, valves, and pericardial structures should be automatically included into quantification. Also, instant multiple frame-by-frame segmentation method is needed to obtain average quantitative parameters in patients with irregular heartbeats.

## Conclusion

We demonstrated that three current prominent deep learning-based fully automated methods are all reliable to perform four-chamber segmentation and quantification of clinical indices without any superiority. However, the performances of these methods were significantly dependent on image quality as well as the number of well annotated training datasets. Additionally, this is the first study to validate the reproducibility of the four cardiac chamber ground-truth annotation itself showing overall excellent reproducibility, but some degree of inter-observer variability still being noted. This study emphasizes that further technical advancement of the fully automated deep learning methods is needed to maintain the clinical performance even in low quality image and specific disease models, not only that, well-designed clinical validation studies are desperately warranted for these technologies to be applied into clinical practice.
